# A case of Libman–Sacks endocarditis in a patient with systemic lupus erythematosus and antiphospholipid syndrome

**DOI:** 10.1093/ehjcr/ytae503

**Published:** 2024-09-16

**Authors:** Adam Bowden, Ronan Walsh, Ross Murphy, Donal Sexton

**Affiliations:** Renal Unit, St James’s Hospital, James’s Street, Dublin 8, Ireland, D08 NHY1; Department of Cardiology, St James’s Hospital, James’s Street, Dublin 8, Ireland, D08 NHY1; Department of Cardiology, St James’s Hospital, James’s Street, Dublin 8, Ireland, D08 NHY1; School of Medicine, Trinity College Dublin, 152-160 Pearse Street, Dublin 2, Ireland, D02 PN40; Renal Unit, St James’s Hospital, James’s Street, Dublin 8, Ireland, D08 NHY1; School of Medicine, Trinity College Dublin, 152-160 Pearse Street, Dublin 2, Ireland, D02 PN40

A 60-year-old male with a history of systemic lupus erythematosus (SLE) with class III lupus nephritis, haemolytic anaemia, and antiphospholipid syndrome (APLS) had his maintenance mycophenolate mofetil (MMF) held due to a recent hospital acquired pneumonia. His warfarin was subsequently held for an elective lumbar puncture to investigate incidental findings on a recent CT brain suggestive of normal pressure hydrocephalus. Seventeen days later, he developed sudden onset left hand weakness with bilateral multifocal infarcts evident on MRI brain. Cardiac telemetry showed a normal sinus rhythm. Transoesophageal echocardiography however revealed small, triangular shaped vegetations on the anterior and posterior mitral valve leaflet tips with associated mitral regurgitation (*Figure 1A–D*). Aside from mild tricuspid regurgitation, the remaining valves appeared normal. Blood cultures were persistently negative. He was restarted on MMF at induction dosages (3 g per day), corticosteroids, and therapeutic anticoagulation; initially with therapeutic low molecular weight heparin followed by warfarin. Transoesophageal echocardiography 25 days later (*Figure 1E–H*) demonstrated complete resolution of the vegetations. The patient was maintained on maintenance dosages of MMF, corticosteroid, and warfarin without recurrence of the endocarditis. Libman–Sacks endocarditis (also called nonbacterial thrombotic endocarditis or marantic endocarditis) is commonly associated with malignancy, SLE, and APLS.^1^ Clinical consequences vary from systemic thrombo-embolization, or valvular dysfunction, to asymptomatic disease and incidental findings at post-mortem. Due to its infrequency, the optimal treatment strategy for Libman–Sacks endocarditis is not well established but predominantly relates to the treatment of the underlying disease.^2,3^ In the case of SLE, this consists of a combination of immunosuppression and anticoagulation. While anticoagulation is first-line treatment in APLS, the addition of immunosuppression may be required in individual cases, particularly in association with catastrophic antiphospholipid syndrome.^1,3^

**Figure 1:**
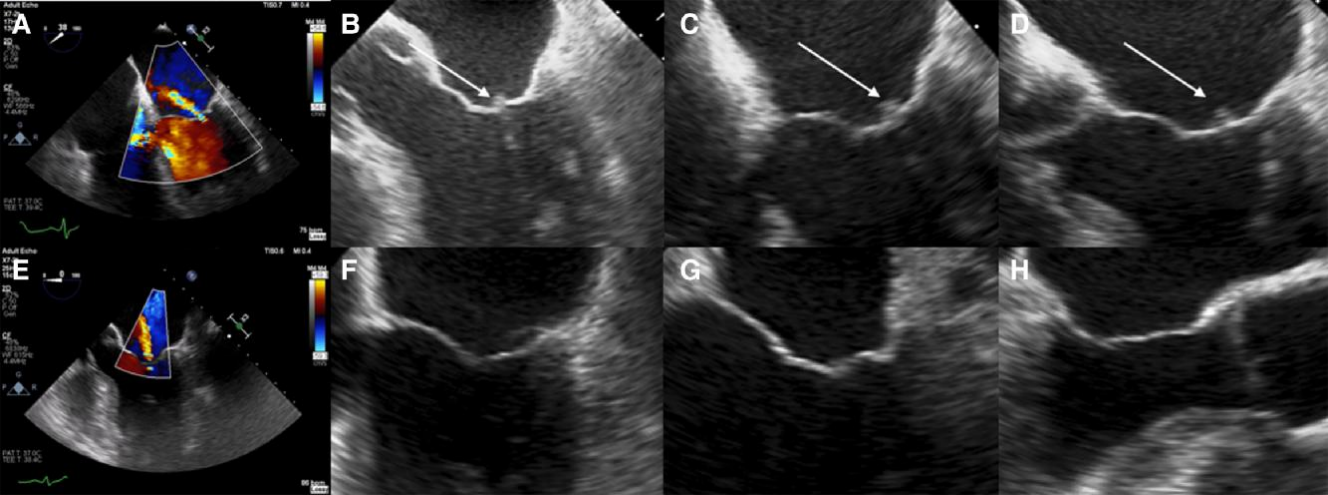
Transoesophageal echocardiogram before (*A–D*) and after treatment (*E–H*). (*A*) Colour flow Doppler showing +2 mitral regurgitation. (*B*) 0° mid-oesophageal two-chamber view of the mitral valve showing a 4 mm vegetation on the posterior and anterior leaflet tips. (*C* and *D*) 38° and 20° mid-oesophageal two-chamber views pre-treatment. (*E*) Colour flow Doppler showing residual 2+ mitral regurgitation. (*F*) 0° mid-oesophageal two-chamber view of the mitral valve showing complete resolution of the vegetation on the posterior leaflet tip. (*G*) 30° mid-oesophageal two-chamber view and (*H*) 128° mid-oesophageal AV long axis view of the mitral valve post-treatment.

**Consent**: The authors confirm that written consent for submission and publication of this case report including images has been obtained from the patient in line with COPE guidance.

## Data Availability

The data underlying this article are available in the article.

## Notes

**Funding:** None declared.

## References

[1] Yoo B-W, Lee S-W, Song JJ, Park Y-B, Jung SM. Clinical characteristics and long-term outcomes of Libman–Sacks endocarditis in patients with systemic lupus erythematosus. Lupus 2020;29:1115–1120. 32536317 10.1177/0961203320930097

[2] Liu J, Frishman WH. Nonbacterial thrombotic endocarditis: pathogenesis, diagnosis, and management. Cardiol Rev 2016;24:244–247. 27501336 10.1097/CRD.0000000000000106

[3] Ibrahim AM, Siddique MS. Libman Sacks endocarditis. Treasure Island (FL): StatPearls; 2021. 30422459

